# Supplementary material for: RNA-FrameFlow: Flow Matching for de novo 3D RNA Backbone Design
Source: ArXiv. 2025 Aug 11:arXiv:2406.13839v4. Originally published 2024 Jun 19. Preprint. [Version 4] (PMC11213149)
Supplement: Supplement 1 [file NIHPP2406.13839v4-supplement-1.pdf]

Appendices

A Additional Experimental Details **16**

A.1 Denoiser Hyperparameters . . . 16

A.2 Upper bound performance of the Self-consistency Pipeline . . . 16

A.3 RhoFold Length Bias . . . 16

A.4 Imputing Non-frame Atoms from Torsion Angles . . . 18

B Ablations **19**

B.1 Composition of Backbone Coordinate Loss . . . 19

B.2 Composition of Auxiliary Loss . . . 19

B.3 Choice of Forward-folding Model . . . 20

B.4 Rotational and All-atom Self-consistency . . . 21

C Additional Results **22**

C.1 Evaluation of MMDiff Samples . . . 22

C.2 Evaluation of Data Preparation Strategies . . . 23

C.3 Comprehensive local evaluation of angular distributions . . . 24

C.4 Measuring All-atom Steric Clashes . . . 25

C.5 Atomic Displacement of Frame Atoms . . . 26

C.6 Modeling Ring Puckering . . . 26

A Additional Experimental Details

A.1 Denoiser Hyperparameters

Category	Hyperparameter	Value
Invariant Point Attention (IPA)	Atom embedding dimension D_h	256
	Hidden dimension D_z	128
	Number of blocks	6
	Query and key points	8
	Number of heads	8
	Key points	12
Transformer	Number of heads	4
	Number of layers	2
Torsion Prediction MLP	Input dimension	256
	Hidden dimension	128
Schedule	Translations (training / sampling)	linear / linear
	Rotations (training / sampling)	linear / exponential
	Number of denoising steps N_T	50

Table 4: Hyperparameters for best performing denoiser model.

A.2 Upper bound performance of the Self-consistency Pipeline

Our self-consistency pipeline to compute *validity* involves inverse and forward folding using gRNAde (Joshi et al., 2025) and RhoFold (Shen et al., 2022). Placing upper bounds on the performance of our RNA backbone design pipeline offers insights into areas of improvement using available open-source tools.

To quantify the total error accumulated in our self-consistency pipeline, and its impact on downstream *validity*, we study the extent to which gRNAde and RhoFold can retrieve the ground truth sequences and structures from the RNAsolo training set. To assess RhoFold's structure prediction performance, we take all ground truth sequences of length 40 – 150 from RNAsolo, forward-fold (FF) them using RhoFold, and compute self-consistency metrics (TM-score, RMSD) by comparing them with the sequences' associated 3D folds. To assess gRNAde's sequence recovery performance, we inverse-fold (IF) 3D backbones from RNAsolo through gRNAde to get 16 likely sequences and pass them to RhoFold for forward-folding.

As shown in the table below, the average self-consistency of the gRNAde-RhoFold pipeline with RNAsolo ground truth backbone structures is 43.7%, close to RNA-FrameFlow's *validity* of 41.0%. This shows us that the generated backbones from RNA-FrameFlow closely retain the validity of RNAsolo backbones and corresponding sequences from gRNAde. In Figure 7, we also show the self-consistency TM-scores per length bins.

Pipeline	Self-consistency (%) ↑	Avg `scTM` ↑	Avg `scRMSD` ↓
RNAsolo + FF only	55.1	0.690	2.804
RNAsolo + IF + FF	43.7	0.663	3.085
RNA-FrameFlow + IF + FF (ours)	41.0	0.641	2.298

A.3 RhoFold Length Bias

We investigate the performance of RhoFold on a representative subset of the training dataset used to train RNA-FrameFlow. Figure 8 shows that RhoFold has a sequence length bias where it predicts accurate structures with low RMSDs (to the ground truth) for specific sequence lengths (like 70, 100, and 120) while predicting poor structures for other lengths. The performance across lengths is disparate and may influence what is considered *valid* in our unconditional generation benchmarks. This affects its efficacy when used in a self-consistency pipeline with the RMSD metric. To minimize the influence of this length bias, we use TM-score for self-consistency because it does not penalize flexible regions as much as RMSD.

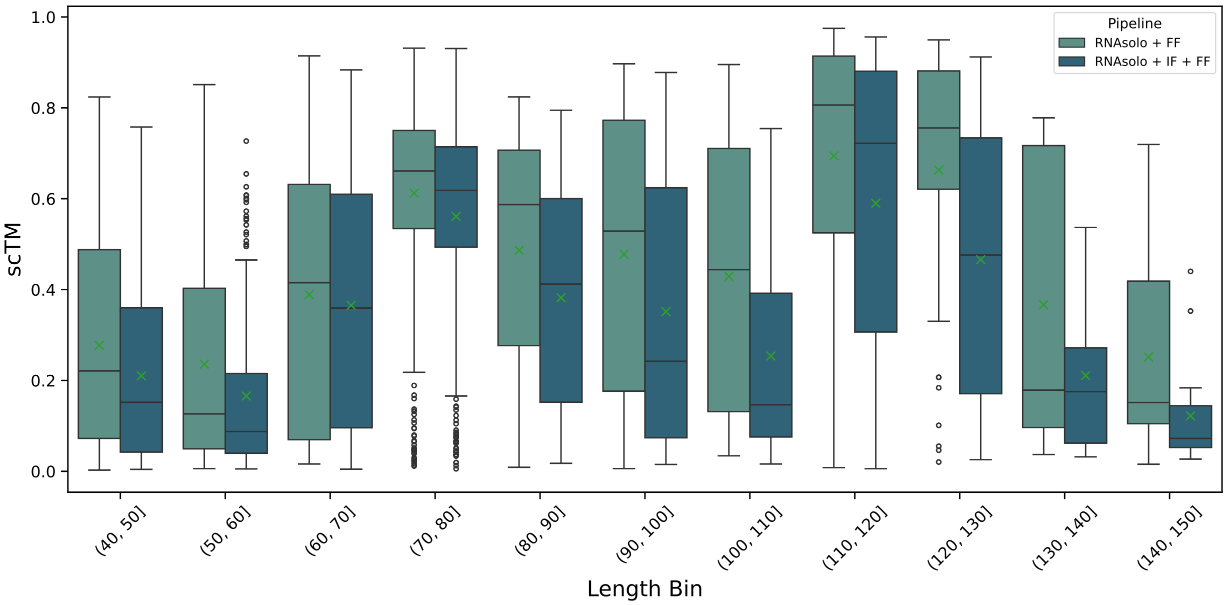

Figure 7: **Self-consistency scores on RNAsolo samples by sequence length**. We observe that generated backbones from RNA-FrameFlow retain the self-consistency of gRNAde-predicted sequences.

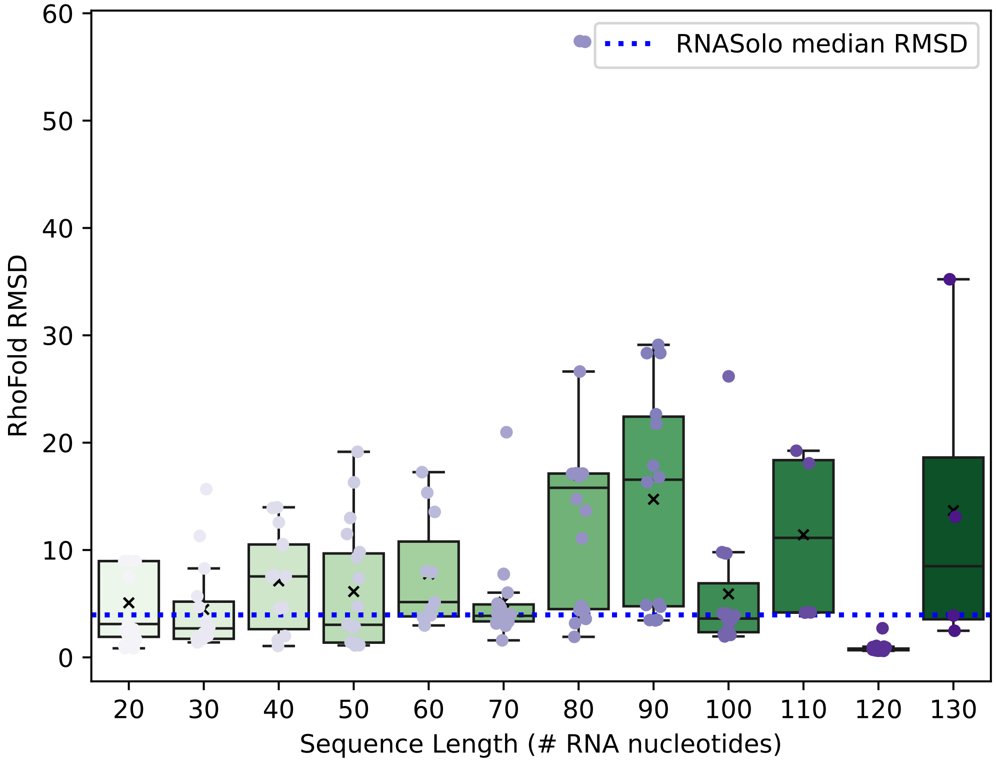

Figure 8: **RhoFold length bias**. The blue dotted line represents the median RMSD of RhoFold predictions to the RNAsolo samples. RhoFold performs well for over-represented sequence lengths in the PDB, and poorly for under-represented sequence lengths.

A.4 Imputing Non-frame Atoms from Torsion Angles

Here, we describe how we autoregressively impute the remaining non-frame atoms using 8 torsion angles $\Phi = \{\phi_1 \rightarrow \phi_8\}$. For a nucleotide n along the generated RNA backbone, we assume we have its final frame $T^{(n)} = (r^{(n)}, x^{(n)})$ obtained from the denoiser's output after N_T diffusion timesteps. Going by our choice of frame $\{C3', C4', O4'\}$, we place non-frame atoms in the following order: $C2', C1', N1/N9, O3', O5', P, OP1, OP2$, each corresponding to its respective $\phi_i \in \Phi$ as shown in Figure 1.

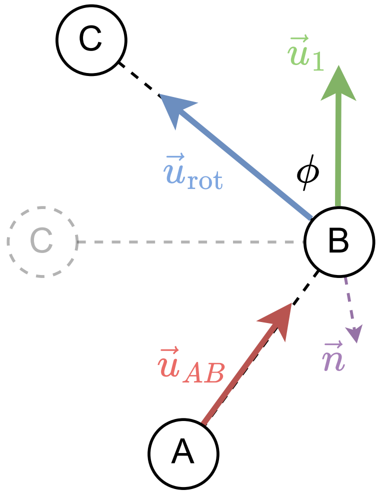

Referring to the figure on the right, suppose we have three atoms A, B, and C with coordinates $(x_A, y_A, z_A), (x_B, y_B, z_B), (x_C, y_C, z_C)$. They are connected by bonds AB and BC denoted by vectors $\overrightarrow{AB} = B - A$ and $\overrightarrow{BC} = C - B$ with lengths $r_{AB} = |\overrightarrow{AB}|$ and $r_{BC} = |\overrightarrow{BC}|$ respectively. To rotate BC around AB by some angle ϕ, we perform the following procedure:

1. Compute unit vector $\vec{u}_{AB} = \frac{\overrightarrow{AB}}{r_{AB}}$ along bond AB by normalizing $\overrightarrow{AB}$.
2. Compute a vector perpendicular to $\vec{u}_{AB}$ by choosing a random normal vector $\vec{n}$ (like $[1,0,0]^T$ or $[0,1,0]^T$) and taking their cross product to get $\vec{u}_1 = \vec{u}_{AB} \times \vec{n}$.
3. Compute the unit vector $\vec{u}_{\text{rot}}$ rotated by ϕ around AB using Rodrigues' rotation formula:
$$\vec{u}_{\text{rot}} = \cos(\phi) \cdot \vec{u}_1 + \sin(\phi) \cdot (\vec{u}_{AB} \times \vec{u}_1) + (1 - \cos(\phi))(\vec{u}_{AB} \cdot \vec{u}_1) \cdot \vec{u}_{AB} \ .$$
4. Compute the coordinates of atom C as follows:
$$[x_C, y_C, z_C] = [x_B, y_B, z_B] + r_{BC} \cdot \vec{u}_{\text{rot}} \ .$$

We use predetermined bond lengths between atoms in the idealized geometry of the Adenine (`A`) nucleotide from OpenComplex (Jingcheng et al., 2022) in the same way Yim et al. (2023b;a) use Alanine for generated protein backbones. We use the following atom triplets and predicted torsion angles to build the all-atom nucleotide, starting from the ribose sugar ring towards the 5′ end (i.e., the phosphate group):

Fixed bond	Non-frame atom	Torsion angle
$C4' - C3'$	$C2'$	ϕ_1
$C4' - O4'$	$C1'$	ϕ_2
$O4' - C1'$	$N9$ (or $N1$)	ϕ_3
$C4' - C3'$	$O3'$	ϕ_4
$C4' - C5'$	$O5'$	ϕ_5
$C5' - O5'$	P	ϕ_6
$O5' - P$	$OP1$	ϕ_7
$O5' - P$	$OP2$	ϕ_8

B Ablations

B.1 Composition of Backbone Coordinate Loss

We also analyze how changing the composition of atoms in the inter-atom losses affects performance. We increase the number of atoms being supervised in the $\mathcal{L}_{\text{bb}}$ loss described above. Aside from the frame comprising $\{C3', C4', O4'\}$, we try two settings with 3 and 7 additional non-frame atoms included in the loss. For the 3 non-frame atoms, we additionally choose $\{C1', P, O3'\}$, and for the 7 non-frame atoms, we choose a superset $\{C1', P, O3', C5', OP1, OP2, N1/N9\}$. We posit the additional supervision may increase the local structural realism, which may further improve validity, as shown in Table 5.

We indeed observe increasing validity as we increase the frame complexity in the auxiliary backbone loss. The minute RMSD contributions from disordered fragments of the RNA may be minimal, accounting for greater likeness to the RhoFold predicted structures, scoring relatively higher `scTM` scores. However, the original frame-only baseline model has better diversity and novelty which we attribute to high local variation in atomic placements. This variation causes two generated structures for the same sequence length to look very different at an all-atom resolution.

**Frame composition in $\mathcal{L}_{\text{bb}}$**	% Validity ↑	Diversity ↑	Novelty ↓
Frame only (baseline)	41.0	**0.62**	**0.54**
Frame and 3 non-frame	45.0	0.28	0.79
Frame and 7 non-frame	**46.7**	0.35	0.85

Table 5: Ablating composition of backbone loss $\mathcal{L}_{\text{bb}}$. Supervising more non-frame atoms improves validity but worsens diversity and novelty. Best result per column is highlighted.

B.2 Composition of Auxiliary Loss

We ablate the inclusion of different auxiliary loss terms that guide our $SE(3)$ flow matching setup; results are in Table 6. Although, there is an increase in EMD for bond distances as we remove distance-based losses like backbone coordinate loss $\mathcal{L}_{\text{bb}}$ and all-to-all pairwise distance loss ($\mathcal{L}_{\text{dist}}$). However, we also observe the model still learns realistic distributions despite removing different loss terms, indicating that each loss makes up for the absence of the other. Moreover, the best model still uses all losses with any removal causing a drop in validity. Further inspecting the samples from the models without each loss term reveals structural deformities at the all-atom level. Figure 9 shows such artifacts resulting from not enforcing geometric constraints through explicit losses.

$\mathcal{L}_{\text{bb}}$	$\mathcal{L}_{\text{dist}}$	$\mathcal{L}_{SO(3)}$	EMD (distance) ↓	EMD (angles) ↓	EMD (torsions) ↓	% Validity ↑
✓	✓	✓	**0.17**	**0.11**	**2.36**	**41.0**
✓		✓	0.18	0.14	3.85	35.0
✓	✓		0.23	0.11	3.72	13.3
	✓	✓	0.18	0.18	3.59	16.7

Table 6: Ablations of loss terms on Earth Mover's Distance scores for structural measurements compared to ground truth measurements from the training set. The first row corresponds to the baseline model. Distance-based losses like the backbone coordinate loss ($\mathcal{L}_{\text{bb}}$) and all-to-all pairwise distance loss ($\mathcal{L}_{\text{dist}}$) are necessary to learn geometric properties like bond distances adequately.

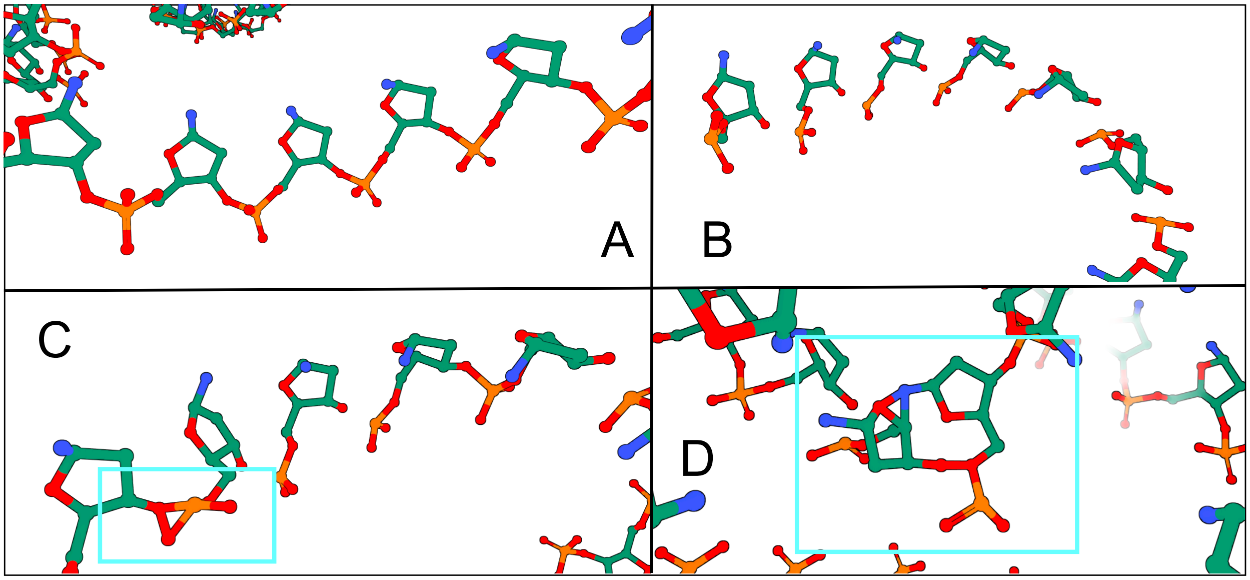

Figure 9: Not including auxiliary losses causes structural deformities in generated RNAs. **(A)** RNA backbone from our baseline model with expected adherence to bonding between nucleotides. **(B)** Not including the rotation loss $\mathcal{L}_{SO(3)}$ causes nucleotides to have random orientations, preventing them from connecting contiguously. **(C)** Not including the backbone atom loss $\mathcal{L}_{\text{bb}}$ places intra-residue atoms too close to one another resulting in bonds that should not exist. **(D)** Not including the all-to-all pairwise distance loss $\mathcal{L}_{\text{dist}}$ causes adjacent frames to fuse and loses contiguity, especially along helices and loops.

B.3 Choice of Forward-folding Model

In our work, we rely on RhoFold (Shen et al., 2022) to forward fold the inverse-folded sequences from gRNAde. Here, we reperform our evaluation from Section 4.1 with Chai-1 (Boitreaud et al., 2024), a recent open-source structure prediction model with results similar to AlphaFold2, replacing RhoFold in the self-consistency pipeline in Figure 2. We do not use MSAs for Chai-1. We do not observe any significant difference in self-consistency distributions: for RNA-FrameFlow$_{\text{RhoFold}}$, we report a *validity* of 41.0% while RNA-FrameFlow$_{\text{Chai-1}}$ gives a *validity* of 39.5%.

Recent benchmarks (Tarafder et al., 2024) also observe that existing RNA structure prediction tools like RhoFold, RF2NA (Baek et al., 2022b), and trRosettaRNA (Wang et al., 2023) perform similarly due to similarities in their architectures and training data. For 600 generated backbones from RNA-FrameFlow, we get 8 predicted sequences from gRNAde, giving us 4800 predicted backbones from RhoFold and Chai-1. We compare this `scTM` distribution across predicted structures in Figure 10.

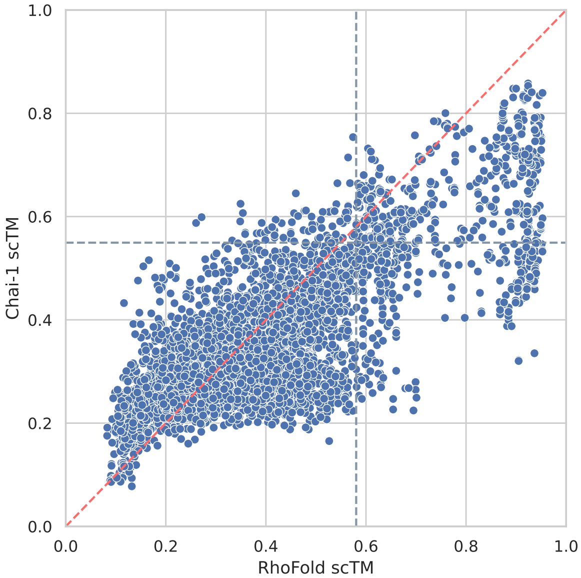

Figure 10: **Correlation between RhoFold and Chai-1 `scTM` scores.** Horizontal and vertical dotted lines denote the median `scTM` score from each method across all samples.

B.4 Rotational and All-atom Self-consistency

Our self-consistency pipeline currently computes TM-score and RMSD with $C4'$ coarse graining, reflecting performance only along the translational component. However, our frames not only comprise the $C4'$ atom but the $C3'$ and $O4'$ atoms as well, forming a rotational component. Factoring in this *rotational* component into the self-consistency metrics would offer a clearer picture of RNA-FrameFlow's ability to precisely orient frames. In Table 7, we report `scRMSD` statistics over our generated backbones. We observe that the `scRMSD` values from RNA-FrameFlow samples correlate positively with the self-consistency scores in Figure 3 (left). For sequence lengths with high % *validity*, we see relatively lower `scRMSD` values and higher `scTM` scores.

**Sequence length**	**Median `scRMSD`** ↓	**Mean `scRMSD`** ↓	**Std. Dev. `scRMSD`** ↓
40	3.81	6.01	4.15
50	3.66	9.36	10.6
60	5.12	9.36	9.98
70	3.74	6.88	8.85
80	6.06	8.43	5.95
90	10.38	11.71	6.74
100	13.27	13.54	6.65
110	14.36	13.33	6.43
120	1.89	3.034	3.89
130	17.45	16.23	5.82
140	11.17	13.06	4.90
150	20.28	20.29	4.67

Table 7: Statistics of rotational `scRMSD` across sequence lengths. We observe RNA-FrameFlow orients frames realistically, correlating positively with reported scTM in Section 4.1 across sequence lengths.

RNA-FrameFlow generates backbones at an all-backbone-atom granularity. We additionally compute `scRMSD` across all 13 nucleotide atoms in Table 8. We similarly observe RNA-FrameFlow can generate realistic fine-grained nucleotides: for sequence lengths with relatively higher % *validity*, we see lower all-atom `scRMSD` values.

**Sequence length**	**Median `scRMSD`** ↓	**Mean `scRMSD`** ↓	**Std. Dev. `scRMSD`** ↓
40	4.22	6.36	3.88
50	4.05	9.73	10.48
60	5.41	9.65	9.85
70	4.19	7.27	8.71
80	6.20	8.70	5.78
90	10.53	11.87	6.65
100	13.26	13.65	6.57
110	14.39	13.48	6.25
120	2.82	3.85	3.67
130	17.47	16.31	5.74
140	11.29	13.19	4.85
150	20.29	20.32	4.64

Table 8: Statistics of all-backbone-atom `scRMSD` across sequence lengths. We observe RNA-FrameFlow generates realistic fine-grained nucleotides, correlating positively with reported EMD scores for local structural descriptors in Table 2 across sequence lengths.

C Additional Results

C.1 Evaluation of MMDiff Samples

Here, we document global and local metrics from samples generated by MMDiff. MMDiff has a validity score of 0.0% as all the samples have a poor `scTM` score below the 0.45 threshold to the RhoFold predicted backbones. Even though none of the samples are valid, we show the average `pdbTM` scores for the samples, which are trivially low as there are no structures from the PDB that match them due to poor quality.

While MMDiff's samples locally resemble RNA structures given realistic, manual inspection reveals multiple chain breaks and disconnected floating strands, resulting in 0.0% validity. In Figure 12 (Subplot 1), we see inter-residue $C4'$ distances slightly varying, causing the chain breaks and clashes. Furthermore, the Ramachandran plot in Figure 12 (Subplot 4) reveals a more complex angular distribution than found in the training set, which may be a consequence of excessively folded regions or substructures that may have folded in on themselves.

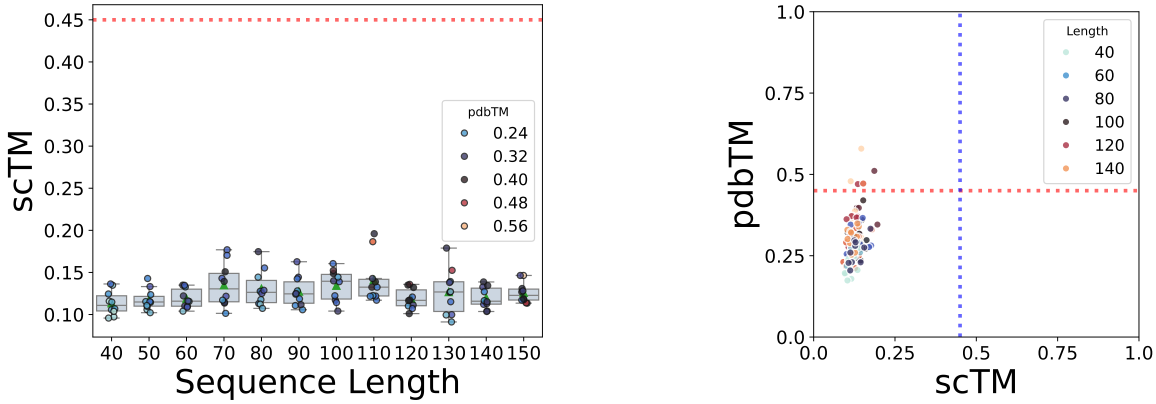

Figure 11: Validity and novelty of retrained MMDiff's top-10 generated backbones. **(Left)** `scTM` of backbones of lengths 40-150 with the mean and spread of `scTM` for each length. **(Middle)** Scatter plot of self-consistency TM-score (`scTM`) and novelty (`pdbTM`) across lengths. Vertical and horizontal dotted lines represent TM-score thresholds of 0.45. Overall, MMDiff retrained on our training set does not generate realistic RNA structures.

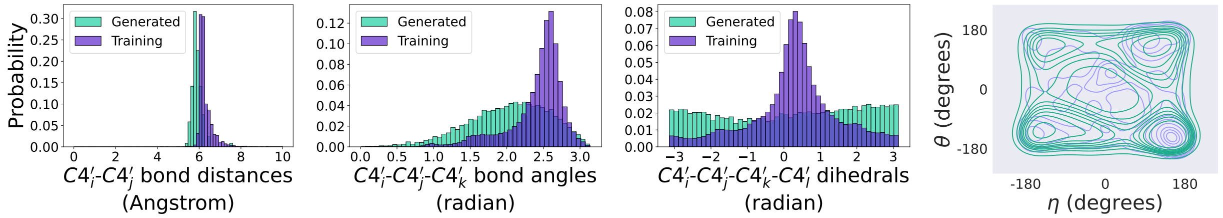

Figure 12: **Structural measurements** from samples generated by MMDiff. **(Subplots 1-3)** Left: histogram of inter-nucleotide bond distances in Angstrom. Middle: histogram of bond angles between nucleotide triplets. Right: histogram of torsion (dihedral) angles between every four nucleotides. **(Subplot 4)**: RNA-centric Ramachandran plot of structures from the training set (purple) and MMDiff's generated backbones (green).

C.2 Evaluation of Data Preparation Strategies

We include global evaluation metrics for the two data preparation strategies presented in the main text, namely structural clustering and cropping augmentation.

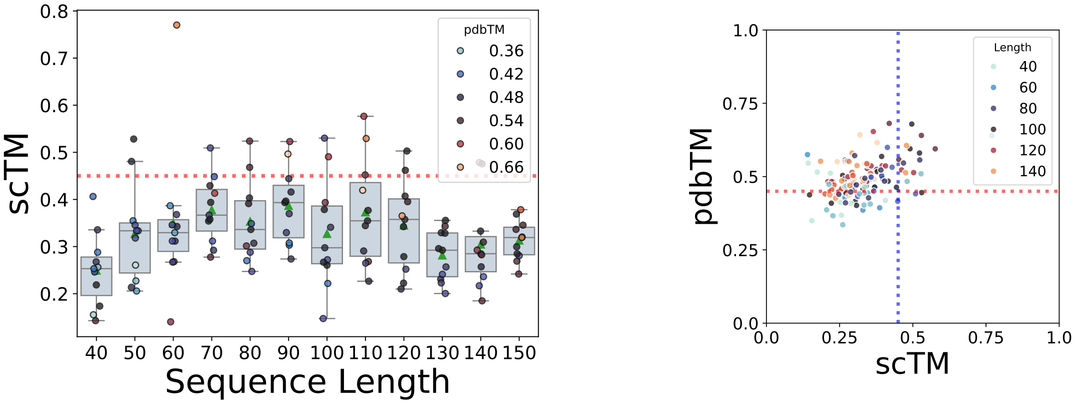

Figure 13: Validity and novelty of top-10 generated backbones from the model trained with only structural clustering. **(Left)** `scTM` of backbones of lengths 40-150 with the mean and spread of `scTM` for each length. **(Middle)** Scatter plot of self-consistency TM-score (`scTM`) and novelty (`pdbTM`) across lengths. Vertical and horizontal dotted lines represent TM-score thresholds of 0.45.

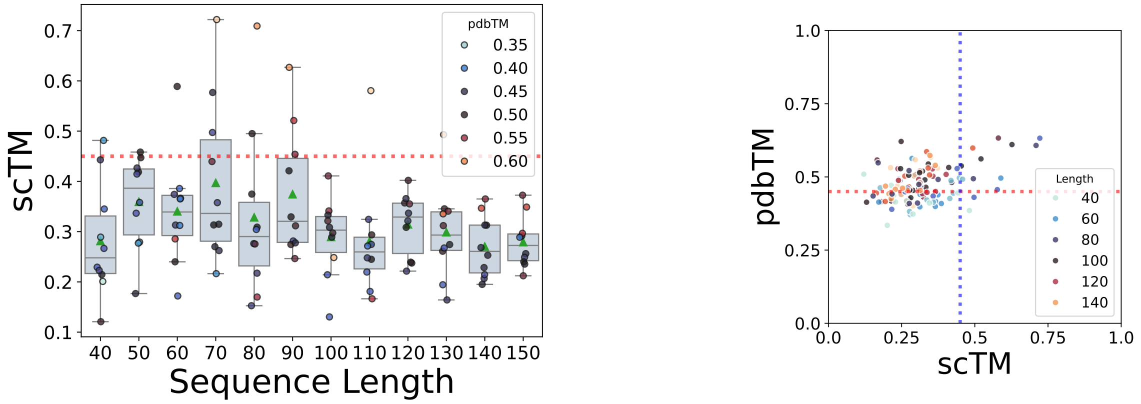

Figure 14: Validity and novelty of top-10 generated backbones from the model trained with structural clustering and cropping. **(Left)** `scTM` of backbones of lengths 40-150 with the mean and spread of `scTM` for each length. **(Middle)** Scatter plot of self-consistency TM-score (`scTM`) and novelty (`pdbTM`) across lengths. Vertical and horizontal dotted lines represent TM-score thresholds of 0.45.

C.3 Comprehensive local evaluation of angular distributions

Following the empirical structural analysis of RNA by Gelbin et al. (1996), we compare local bond angle distributions among triplets of atoms from the generated backbones. We sample 50 all-atom backbones for each sequence length in $[70, 90, 110, 130, 150]$, sieve out the *valid* samples, and extract relevant bond angles. As shown in Figure 15, we observe that RNA-FrameFlow can retrieve angular distributions between distant and nearby atoms in the nucleotides, providing preliminary evidence that modern protein design models are sufficiently expressive to model RNA tertiary structure.

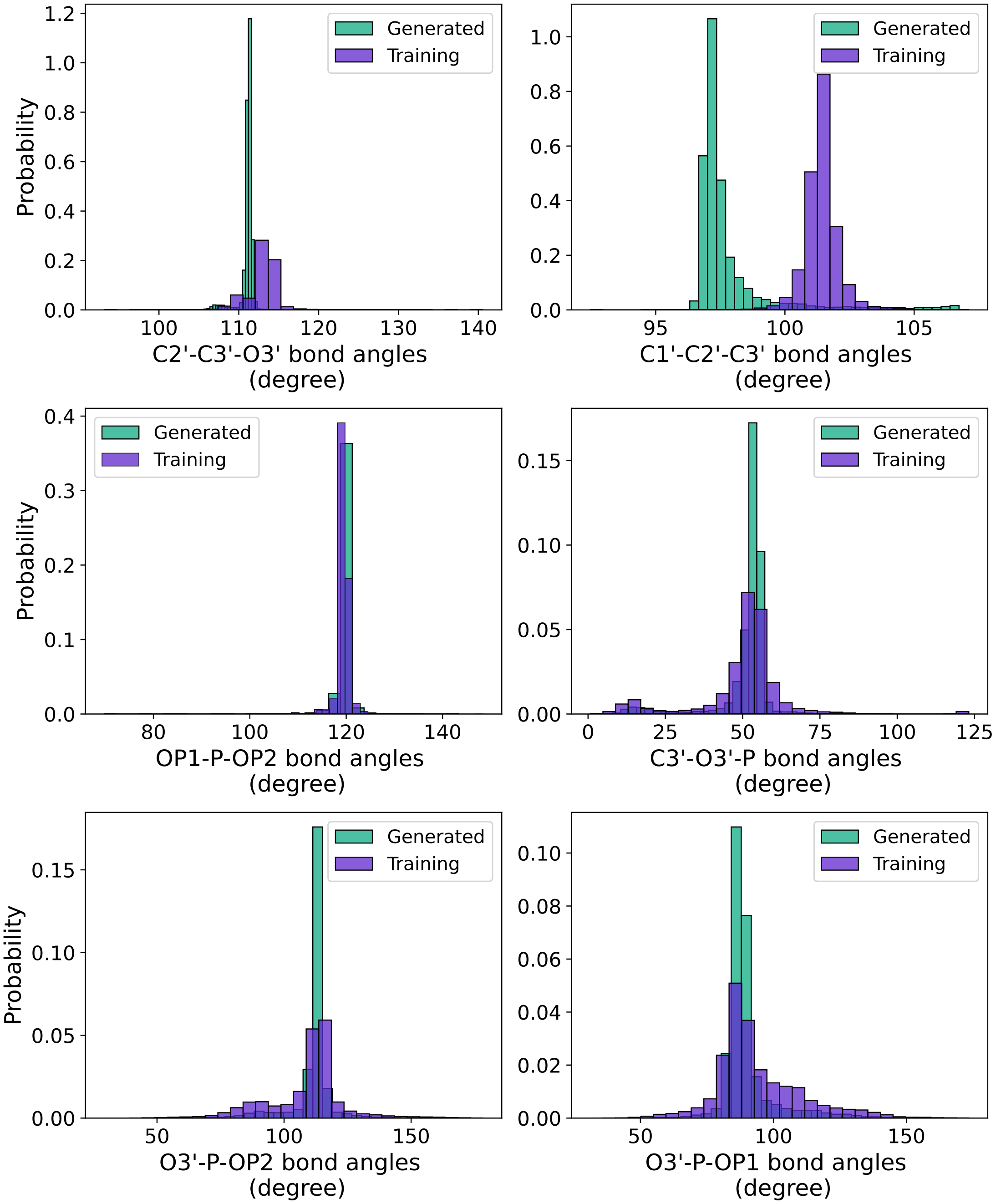

Figure 15: **Bond angle distributions between triplets of atoms**. We select these atomic triplets from the empirical study of RNA's 3D geometry by Gelbin et al. (1996).

C.4 Measuring All-atom Steric Clashes

We compare the *all-atom-level* steric clashes between filtered RNAsolo samples used for training and the generated backbones from RNA-FrameFlow. We say two *unbonded* atoms i, j clash if the distance between them r_{ij} is within a threshold d_{steric}:

$$d_{\text{steric}} = \mathrm{v}_i + \mathrm{v}_j - 0.6 \tag{11}$$

$$\mathbb{I}_{ij} = \begin{cases} 1 & r_{ij} \leq d_{\text{steric}} \\ 0 & \text{otherwise} \end{cases} \tag{12}$$

$$\#\text{ clashes } = \sum_{i,j} \mathbb{I}_{ij}\ . \tag{13}$$

Here, $\mathrm{v}_i, \mathrm{v}_j \in \mathbb{R}$ are the Van der Waals (VdW) radius of the atoms i, j in Angstrom. Based on its identity, each atom has its own VdW radius which we factor into our computation. We leave a generous tolerance of 0.6 Å (corresponding to half the Hydrogen atom's VdW radius of 1.20 Å) to account for random deviations in atomic placements. We ignore Phosphodiester and Glycosidic bonds when computing clashes because the covalent radius is smaller than the VdW radius. As nucleotides are constructed using idealized bonds, there may be fewer inter-nucleotide clashes, resulting in fewer clashes for RNA-FrameFlow backbones.

In Figure 16, we compare the steric clashes across sequence length bins. We observe that RNA-FrameFlow generates backbones that have a similar distribution of inter-atom steric clashes as samples from RNAsolo. We also include *validity* for each sequence length bucket. We see that samples from certain sequence lengths (like 70, 80, 120) contain relatively fewer steric clashes across samples within that length bucket since they are over-represented in RNAsolo. This means RNA-FrameFlow might be better at recapitulating atomic positions for such lengths than others. The steric clashes are normalized by the number of heavy atoms in the molecules, giving us steric clashes per 100 atoms. For the RNAsolo samples, we see 10.03 ± 1.52 clashes per 100 atoms while our generated backbones have 25.55 ± 5.43 clashes per 100 atoms.

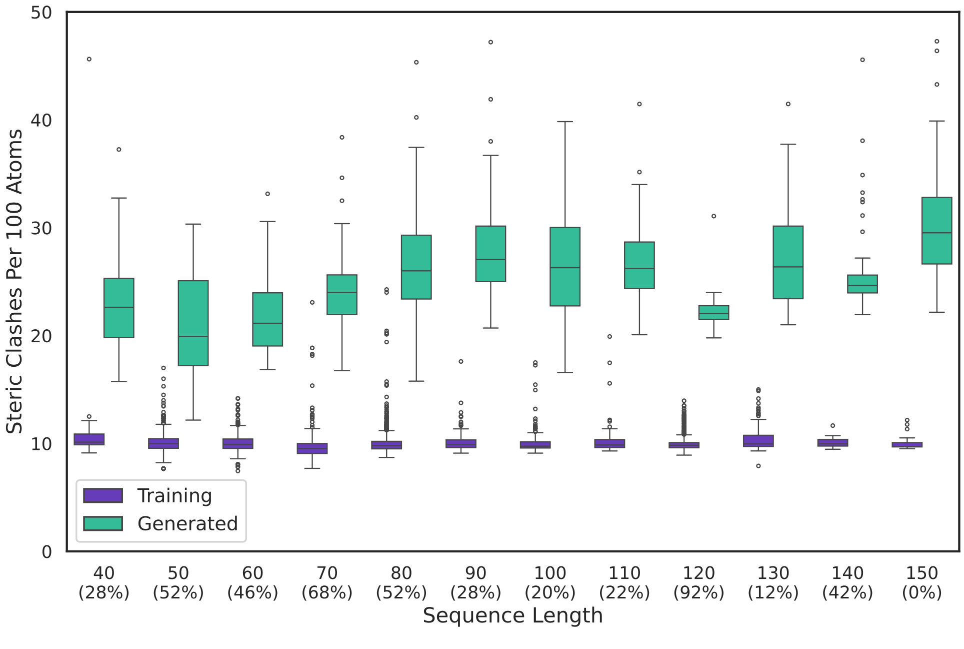

Figure 16: **All-atom steric clashes by sequence length**. We observe a similar number of steric clashes between training and generated backbones across sequence lengths. We include the (% *validity*) for generated samples from each sequence length below the length labels along the horizontal axis.

C.5 Atomic Displacement of Frame Atoms

To further motivate our choice of frame atoms, we examine the B-factor of each atom provided in the RNAsolo PDB files. B-factor is a measure of atomic displacement, i.e., how much an atom *wiggles* in its place when the structure is undergoing X-ray crystallography or Cryo-EM. Empirically, we observe our frame construction $\{C4', C3', O4'\}$ collectively deviates the least compared to the frame construction in RF2NA (Baek et al., 2022b), $\{P, OP1, OP2\}$. We report this in Table 9 below:

Atom	Mean B-factor (Å^2) ↓	Median B-factor (Å^2) ↓
C4'	111.44	84.84
C3'	111.74	85.41
O4'	111.22	84.86
P	114.03	88.69
OP1	113.46	87.56
OP2	112.35	86.11

Table 9: Statistics of B-factor values (atomic displacement) of nucleotides from 500 random samples in RNAsolo. We observe our frame construction $\{C4', C3', O4'\}$ experiences lower collective spatial uncertainty compared to alternate frame constructions such as $\{P, OP1, OP2\}$.

C.6 Modeling Ring Puckering

Puckering refers to non-planar deformations or contortions of the ribose sugar ring in RNA comprising the atoms $\{C1', C2', C3', C4', O4'\}$. Instead of forming an ideal flat plane, the ring atoms alternately move above and below the ideal plane to relieve steric strain (see Figure 17 **(A)**). The five atoms collectively define five out-of-plane torsion angles: ν_0 $(C4' - O4' - C1' - C2')$, ν_1 $(O4' - C1' - C2' - C3')$, ν_2 $(C1' - C2' - C3' - C4')$, ν_3 $(C2' - C3' - C4' - O4')$ and ν_4 $(C3' - C4' - O4' - C1')$. For a nucleotide along the backbone, these angles define *Cremer–Pople pseudo-rotations* parameterized by a phase angle P, that locates which atom is displaced towards the *endo* position (i.e., above the flat plane), and an amplitude τ_m, that quantifies the maximum magnitude of displacement. Tracking these quantities provides insights into the conformational diversity and interactions of RNA. Specifically, $C2'$-endo and $C3'$-endo puckering are known to mediate these behaviours. Several riboswitches, ribozymes, and RNA-protein interfaces exploit such *endo* orientations of ribose atoms as a trigger mechanism during molecular binding (Setlik et al., 1995; Salter et al., 2006).

In naturally-ocurring RNA (e.g., RNAsolo), the phase angle P distribution is sharply bimodal with a dominant $C3'$-endo near $0°$ and a smaller $C2'$-endo near $180°$. The puckering amplitude τ_m forms a narrow band near $32° - 38°$ (0.55-0.66 rad) (Shi et al., 2020; Harp et al., 2022). In Figure 17 **(B)**, we demonstrate that RNA-FrameFlow captures key ring puckering motions, with similar distributions for P and τ_m.

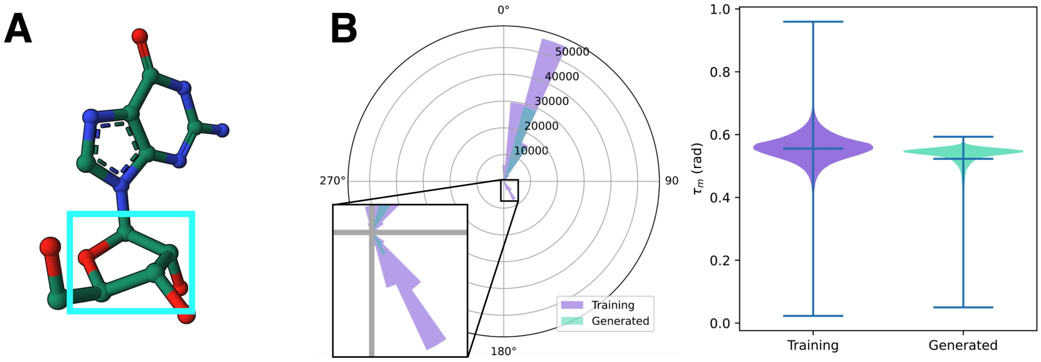

Figure 17: **Analysis of ring puckering motions.** **(A)** Puckering occurs when atoms comprising the ribose sugar ring move above (*endo*) or below (*exo*) the ideal flat plane (cyan box). **(B)** Rose chart of phase angle P distribution (left) and violin plot of amplitude τ_m distribution (right). RNA-FrameFlow (green) generates RNA backbones that capture an appropriate degree of puckering seen in natural RNA (purple).
